# A case for hybrid BCIs: combining optical and electrical modalities improves accuracy

**DOI:** 10.3389/fnhum.2023.1162712

**Published:** 2023-06-07

**Authors:** Rand Kasim Almajidy, Soheil Mottaghi, Asmaa A. Ajwad, Yacine Boudria, Kunal Mankodiya, Walter Besio, Ulrich G. Hofmann

**Affiliations:** ^1^Faculty of Medicine, University of Freiburg, Freiburg im Breisgau, Germany; ^2^Section for Neuroelectronic Systems, Department of Neurosurgery, Medical Center University of Freiburg, Freiburg im Breisgau, Germany; ^3^Roche Diagnostics Automation Solutions GmbH, Ludwigsburg, Germany; ^4^College of Medicine, University of Diyala, Baqubah, Iraq; ^5^Electro Standards Laboratories, Cranston, RI, United States; ^6^Electrical, Computer and Biomedical Engineering, Kingston, RI, United States

**Keywords:** BCI, monitoring brain activity, NIRS system design, multi-modal BCI, EEG, classification, matching pursuit, LASSO

## Abstract

Near-infrared spectroscopy (NIRS) is a promising research tool that found its way into the field of brain-computer interfacing (BCI). BCI is crucially dependent on maximized usability thus demanding lightweight, compact, and low-cost hardware. We designed, built, and validated a hybrid BCI system incorporating one optical and two electrical modalities ameliorating usability issues. The novel hardware consisted of a NIRS device integrated with an electroencephalography (EEG) system that used two different types of electrodes: Regular gelled gold disk electrodes and tri-polar concentric ring electrodes (TCRE). BCI experiments with 16 volunteers implemented a two-dimensional motor imagery paradigm in off- and online sessions. Various non-canonical signal processing methods were used to extract and classify useful features from EEG, tEEG (EEG through TCRE electrodes), and NIRS. Our analysis demonstrated evidence of improvement in classification accuracy when using the TCRE electrodes compared to disk electrodes and the NIRS system. Based on our synchronous hybrid recording system, we could show that the combination of NIRS-EEG-tEEG performed significantly better than either single modality only.

## 1. Introduction

Brain-computer interfacing (BCI) is a technology first mentioned in the 1970s (Vidal, 1973). It is usually thought to employ non-invasive brain monitoring techniques to recklessly collect brain activity data (Koo et al., 2015) and aims to provide people in need with a new communication method (Min et al., 2010). It may form a bridge between brains and the outside world bypassing the classical communication channels (Choi et al., 2017) with the help of advancements in technology (Rashid et al., 2020), such as software (Rao and Scherer, 2013), signal processing (Ramadan and Vasilakos, 2017; Wierzgała et al., 2018) as well as enhanced sensors (Martini et al., 2020).

Both sophisticated hardware and software are needed to create a BCI system as it is supposed to capture seemingly noisy brain activity and translate it into an intended action (Khan et al., 2020). The software part mandates signal preprocessing [e.g., filter motion artifacts (Aggarwal and Chugh, 2019)], feature extraction [e.g., matching pursuit (Mallat and Zhang, 1993)], feature selection [e.g., least absolute shrinkage and selection operator (Tay et al., 2021)], and classification (Spezialetti et al., 2018). Nowadays machine learning approaches may be used to that end as well (Rasheed, 2021).

Utilizing more than one non-invasive brain monitoring technique on the same human scalp at the same time asks for so-called hybrid devices and is a relatively new direction to enhance BCI performance (Pfurtscheller et al., 2010; Fazli et al., 2012; Hong et al., 2018; Shin et al., 2018). To this end, we employed a commercially available EEG amplifier and signals from a special electrode, the tri-polar concentric ring electrode (TCRE) (Besio W. et al., 2006). We integrated the EEG system with a novel near-infrared spectroscopy (NIRS) system (Almajidy et al., 2020) to conduct 2D BCI experiments with a group of volunteers. Although NIRS systems are available commercially, we were inspired with the constant efforts to create new EEG systems and electrodes with enhanced performance and lower cost and decided to create a low cost portable system in an effort to make a portable dual modality more affordable. That way we were able to quantify the contribution of different modalities and their combinations.

## 2. Materials and methods

### 2.1. Data acquisition systems

The novel NIRS system (Almajidy et al., 2014) (see Supplementary Figure 1) feature an Atmel Microcontroller board (ATmega1280/V, USA) to control detector optodes and synchronize the NIR LED sources’ off/on period. It sent the collected data to a laptop at a 22 Hz sampling frequency and controls constant current sources to supply the optodes with a stable current. MATLAB (The MathWorks MATLAB R2022a) scripts in the connected laptop were used to achieve signal preprocessing, filtering, and analysis. The NIRS system casing (which houses the system electronics), sensor casings (which house the detectors’ preamplifier circuits), and the sensor holder rings (which were attached to the cap) were designed with Solid Edge software (Siemens Industry Software GmbH, Germany) and 3D printed with a simple 3D printer (Replicator2, MakerBot Industries, USA) using PLA filament (see Supplementary Figure 1).

**FIGURE 1:**
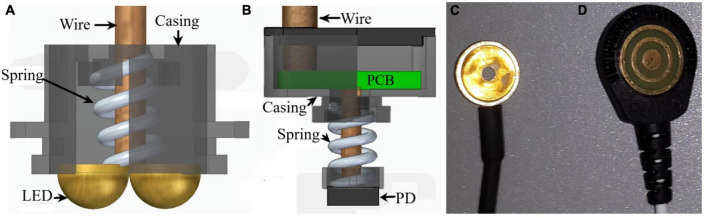
Sensors used for data collection. **(A)** Light source (LED) of the NIRS system, **(B)** its photodiode (PD) detector, **(C)** regular gelled Au disk electrode and **(D)** a TCRE electrode.

Near-infrared spectroscopy optodes (sources/detectors, see Figure 1) feature eight NIR detectors (BPW34-B, OSRAM Opto Semiconductors GmbH, Germany). These photodiodes (PD) had a spectral range of sensitivity between 350–1100 nm, a radiant sensitive area of 7.45 mm^2^, and a 25 ns short switching time. Each detector is attached to a spring to gently press it to the scalp’s skin (see Figure 1).

**FIGURE 2:**
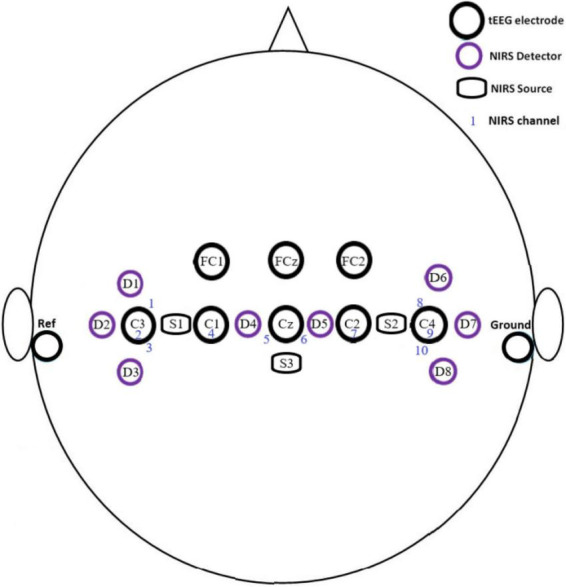
Montage of electrodes, sources, and detectors of the hybrid BCI system during off- and online experiments.

Our NIRS optodes also have three NIRS dual sources, comprised of three 850 nm photodiodes (TSHG6400, Vishay Semiconductors, USA) and three 770 nm photodiodes (MTE1077N1-R, Marktech Optoelectronics, USA). Arranging NIRS sources and detectors in a commercial cap (NIRScap, NIRx Medical Technologies, LLC) forms ten NIRS channels (see Figure 2 for the montage used during experiments, more channels can be created with different alignments). Optode holder rings are arranged on the cap to separate the source from the detector by 3 cm (SDS) to allow light penetration into the cortical region of interest (see Figure 2). The complete NIRS system is versatile, small, and portable (size = 11 cm × 8.5 cm × 3.7 cm) weighing about ∼160 g (including sensors ∼ 390 g). Material price for the system sums to about €1,470.

Two-disk electrodes and eight Tri-Polar Concentric Ring Electrodes (TCRE, CREmedical, USA) connected to a commercial g.USB amplifier (g.tec GmbH, Schiedlberg, Austria) are used to collect scalp potential signals. The TCRE is thought to have a higher local sensitivity and spatial resolution as compared to other electrodes (Besio W. G. et al., 2006; Besio W. et al., 2006). Signals measured by the TCRE outer ring provide an emulated EEG (eEEG) equivalent to a regular disk EEG electrode (Makeyev et al., 2013; Zhu et al., 2014). Hence in this paper, the signal extracted from all three rings (which is processed to a single signal) is denominated tEEG while the signal collected from the TCRE outer ring falls in the category EEG. This peculiarity enables us to compare the performance of both data types during BCI experiments. To enhance EEG data spatial resolution, the scalp surface Laplacian is employed. The second spatial derivative of the surface Laplacian was shown to improve the high spatial frequency components of the brain activity near the electrode (Babiloni et al., 1995). Besio et al. (Besio W. G. et al., 2006; Besio et al., 2008) used a special sensor namely the TCRE electrode (Besio W. G. et al., 2006; Besio et al., 2011) to measure the Laplacian. The signal measured by this sensor is calculated by: [16 * (V_*m*_ − V_*d*_) − (V_*o*_ − V_*d*_)] where V_*m*_, V_*d*_, V_*o*_ are the voltage of the middle ring, central disc, and outer ring of the TCRE electrode, respectively. The EEG signal collected by this electrode is called tEEG by Besio et al. (Besio W. G. et al., 2006; Besio et al., 2008). When compared to disc electrode signals; tEEG signals’ spatial resolution, signal-to-noise ratio, and mutual information are improved by approximately three, four, and twelve times, respectively (Koka and Besio, 2007). The main disadvantage of TCRE electrodes is their large surface area (diameter = 1.6 cm) compared to the typical commercial EEG electrodes (diameter = 1 cm) (Ollikainen et al., 2000).

The main disadvantage of TCRE electrodes is their large surface area (diameter = 1.6 cm) compared to the typical commercial EEG electrodes (diameter = 1 cm) (Ollikainen et al., 2000).

TCRE electrodes are mounted on the cap by 3D-printed holding rings following the international 10–20 system. Electrodes are located at C1, C2, C3, C4, Cz, FC1, FC2, and FCz. A disk electrode is located on the right mastoid and another on the left mastoid. The disk electrodes serve as the reference and ground contact, respectively. The sampling frequency of the measured signals is set to 256 Hz; the signals are notch filtered at 60 Hz frequency (because the experiments were done in the United States as the mains frequency is 60 Hz) and band-pass filtered between 0.1 and 70 Hz. The layout map for the EEG/tEEG electrodes and NIRS optodes covers the motor cortex area of the brain (see Figure 2).

### 2.2. Experimental paradigms

A motor imagery (MI) BCI paradigm was used during the experiments. This paradigm is employed by many researchers (McAvinue and Robertson, 2008; Wierzgała et al., 2018; Khan et al., 2020) and means that the subject will imagine moving his/her hands/feet rather than actually moving them. During the experiments, we input tEEG and EEG data into BCI2000 software (Schalk et al., 2004). The software provided the experiment’s cue (on a 23.8 inch LCD screen 1.5 m from the subject) and showed the subject’s feedback on their performance during online sessions.

The subjects performed the experiments in a dimly lit lab while comfortably seated. To minimize motion artifacts, they were asked to avoid moving. Sixteen subjects participated in the experiments (aged 20–36 years). They all signed an informed consent form before participating. The University of Rhode Island Institutional Review Board (IRB) approved the protocol of the experiments.

**FIGURE 3:**
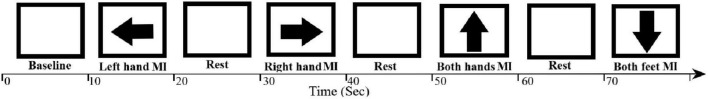
The offline session MI paradigm. The screen presented for each task for 10 s the relevant cue to the subject or was blank during rest between tasks. An arrow pointing to the left, right, down, or up indicates to the subject to start the MI task (imagine left hand move, right hand move, both feet move or both hands move, respectively) while a blank screen indicates a rest period.

The experiments consisted of one offline and one online session. The offline session had five runs; each run consisted of twenty trials. The subjects were asked to imagine one of four MI tasks during each trial corresponding to the cue shown on the screen in front of them. The first task is preceded by a 10-s baseline period. Each task lasts for 10 s and is followed by 10 s of rest. The cue displayed was either an arrow pointing up (imagine moving both hands, *Both-hands MI*), an arrow pointing down (imagine moving both feet, *Both-feet MI*), an arrow pointing left (imagine moving left hand, *Left-hand MI*), an arrow pointing right (imagine moving right hand, *Right-hand MI*), or a blank screen to signal rest (see Figure 3).

The offline session resulted in 100 trials, with 25 trials of each task type. The offline session tasks have fixed and identical task periods, were repeated periodically and the subjects received no feedback about their performance during the task. The session’s purpose is to provide data to train the online classifier. This session was followed by an offline analysis during which the BCI2000 software classifiers were adjusted according to the subject’s performance and prepared for the online session.

**FIGURE 4:**
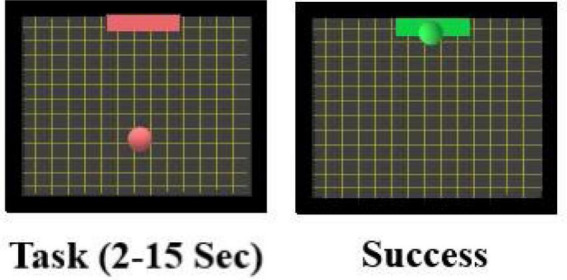
Screenshot during the online BCI MI session. During the task, a red target and a ball is presented. If the subject succeeded in moving the ball into the target by performing the MI tasks (as explained above) the target will change color to green to indicate success.

The online session consisted of five runs with 20 trials each. During these trials, (100 trials per subject) tasks are shown in random order. The task cue given is a red square target appearing at one edge of the screen. A red ball would appear consecutively, and the subject would complete the task by hitting the target based on imagined movements as before. The color of the target and the ball will turn green if the subject accomplishes the task. The task cue starts with 2 s baseline recording followed by 1 s during which the target appears followed by the ball for a period between 2 and 15 s (see Figure 4). This ball’s presentation period depends on the subject’s performance: A successful hit terminates the task otherwise it would last for 15 s. The task is followed by a 1-s feedback displaying either success (green target) or failure (red target). A 15-s rest period (blank screen) completes the trial. The subjects are advised to employ the MI tasks exercised during the offline session. They imagined moving their hands/feet to move the ball up/down and so on.

## 3. Results and discussion

### 3.1. EEG/tEEG data

Collected data were preprocessed to improve the signal-to-noise ratio (SNR). For EEG/tEEG data, we used EEGlab software (Delorme and Makeig, 2004) and bandpass filtered the data between 1 and 48 Hz with a finite impulse response (FIR) filter to minimize the main power crosstalk. We also employed this software to reduce the motion artifacts as well as EOG, and EMG components. We used a MATLAB script to apply a notch FIR filter to reduce interference with our NIRS system.

**FIGURE 5:**
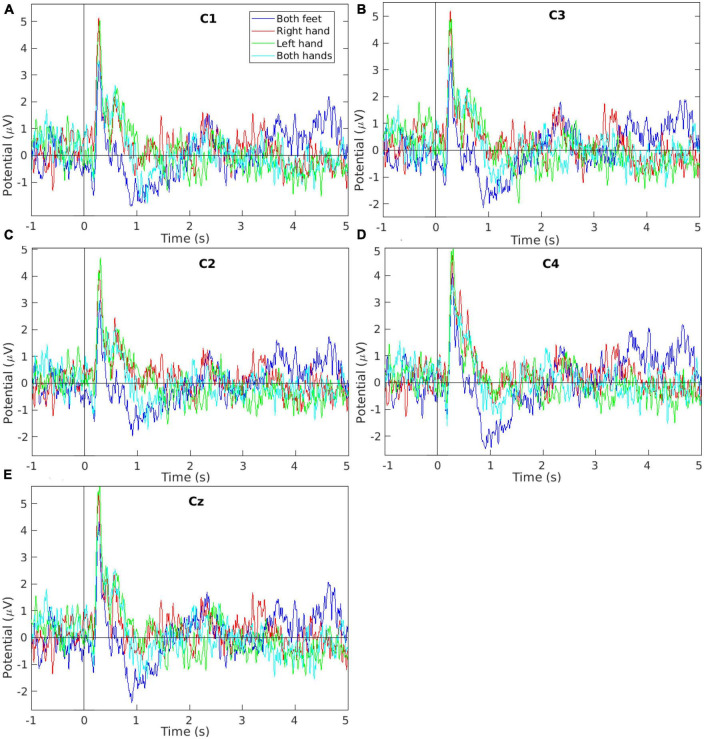
Event-related potential signal grand average of the EEG measurements collected while performing the four motor imagery tasks during the offline session from electrodes **(A)** C1, **(B)** C3, **(C)** C2, **(D)** C4, and **(E)** Cz. The plots show the tasks’ first 5 s (from the total 10 s per task) to improve the visibility as the remaining tasks’ time showed no other peaks.

**FIGURE 6:**
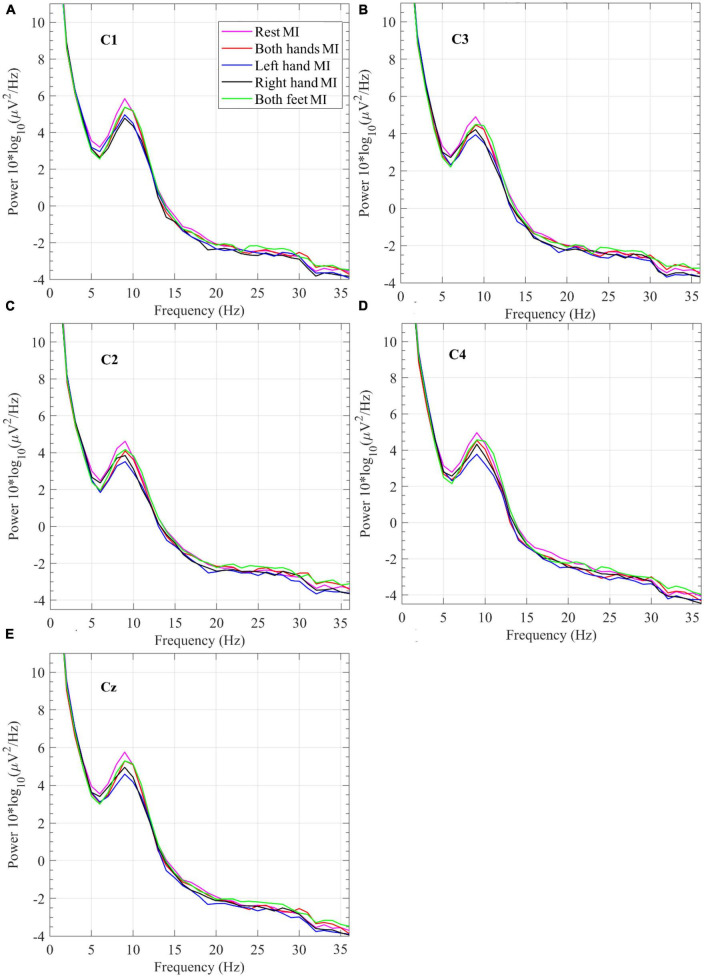
Power spectral density grand average of EEG measurements collected while performing the four motor imagery tasks and Rest during the offline session from electrodes **(A)** C1, **(B)** C3, **(C)** C2, **(D)** C4, and **(E)** Cz.

Data from offline sessions for all subjects were analyzed using EEGlab software to plot event-related potential (ERP) and the power spectral density (PSD). Grand average plots are shown in Figure 5 (ERP grand average) and Figure 6 (PSD grand average). Each task’s grand average is calculated for 400 trials (16 subjects × 5 runs per offline session × 5 task trials per run).

Figure 5 shows the ERP grand averages for EEG data collected from electrodes C1, C2, C3, C4, and Cz. The plots are limited between −1 and 5 s to improve visibility (the original tasks were 10 s each) since no other peaks were seen in the remaining time. In Figures 5A, B, the *Right-hand* MI plot has a higher potential at around 0.3 s compared to other tasks. This is in agreement with the literature (Neuper and Pfurtscheller, 2010; Kaya et al., 2018) as the electrodes C1 and C3 monitor the brain region which controls the right-hand movement. In Figures 5C, D, the *Left-hand* MI plot has a higher potential at around 0.3 s compared to other tasks. This is in agreement with the literature (Romero et al., 2000; Neuper and Pfurtscheller, 2010) as the electrodes C2 and C4 monitor the brain region which controls the left-hand movement. In Figure 5E, the *Both-feet* MI plot has a potential of about 4.2 μV at around 0.3 s. This potential value although not the highest ERP potential in Figure 5E, has the highest *Both-feet* MI potential value in Figure 5. This is in agreement with the literature as the electrode Cz monitors the brain region which controls the feet movements. The lower potential compared to other electrodes is due to the special location of this region within the brain’s central sulcus (Neuper and Pfurtscheller, 2010).

In Figure 6, which displays the plots for the PSD grand averages, the *Rest* plot around 9 Hz shows the highest power. *Right-hand* MI has the lowest power around 9 Hz in data from electrode C1. *Left-hand* MI has the lowest power around 9 Hz in data from electrodes C2 and C4. PSD at the specific brain region of interest is expected to be lower while performing the MI task compared to the rest period.

### 3.2. NIRS data

Data from our NIRS channels was first preprocessed and analyzed by MATLAB scripts. Data was first bandpass filtered between 0.01 and 0.1 Hz by an FIR bandpass to reduce the effect of Mayer waves (Yücel et al., 2016). Data were then smoothed by a 7-point moving average filter. Data from the 10 NIRS channels resulting from the combination of the NIRS sources and detectors (see Figure 2) was collected for the 16 subjects with a total of 400 trials for each task (16 subjects × 5 runs per offline session × 5 tasks per trial run). Only HbO_2_ data were used during NIRS data analysis.

**FIGURE 7:**
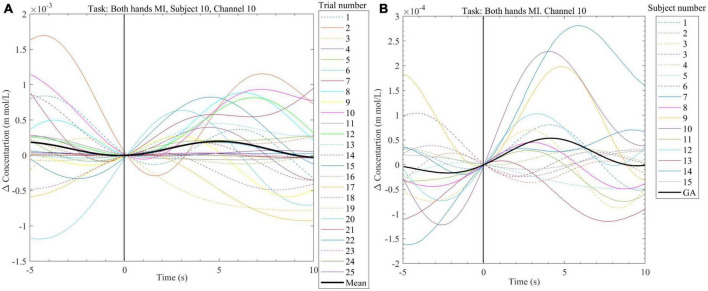
**(A)** Exemplary plot of the change in HbO_2_ concentration calculated from the NIRS signal recorded from subject 10, channel 10 during the offline session. Plots are done for the 25 trials of the *Both-hands* MI task as well as the mean for all the trials (black line). **(B)** Plot of the grand average (black line) change in HbO_2_ and the mean change in HbO_2_ (data from 25 trials per subject) from channel 10. Data were collected from 16 subjects while performing *Both-hands* MI during the offline session.

In Figure 7A, NIRS data from one subject (subject 10) collected from channel 10 was plotted for 25 trials of *Both hand* MI task performed during the offline session, the mean across these trials was also plotted (the black line). Channel 10 monitors the brain area which controls the left hand (see Figure 2). The mean data showed an increase in value with the start of the *Both-hands* MI task. The increase reached its maximum value around 5 s and dropped to 0 at 10 s. The mean data for this subject (subject 10) and the other 15 subjects who participated in the offline sessions were plotted for the same task and the same channel in Figure 7B the grand average (GA) was also plotted (the black line, the peak value around 5 s). The grand average shows an increase during the task which drops in value before the end of the 10 s.

**FIGURE 8:**
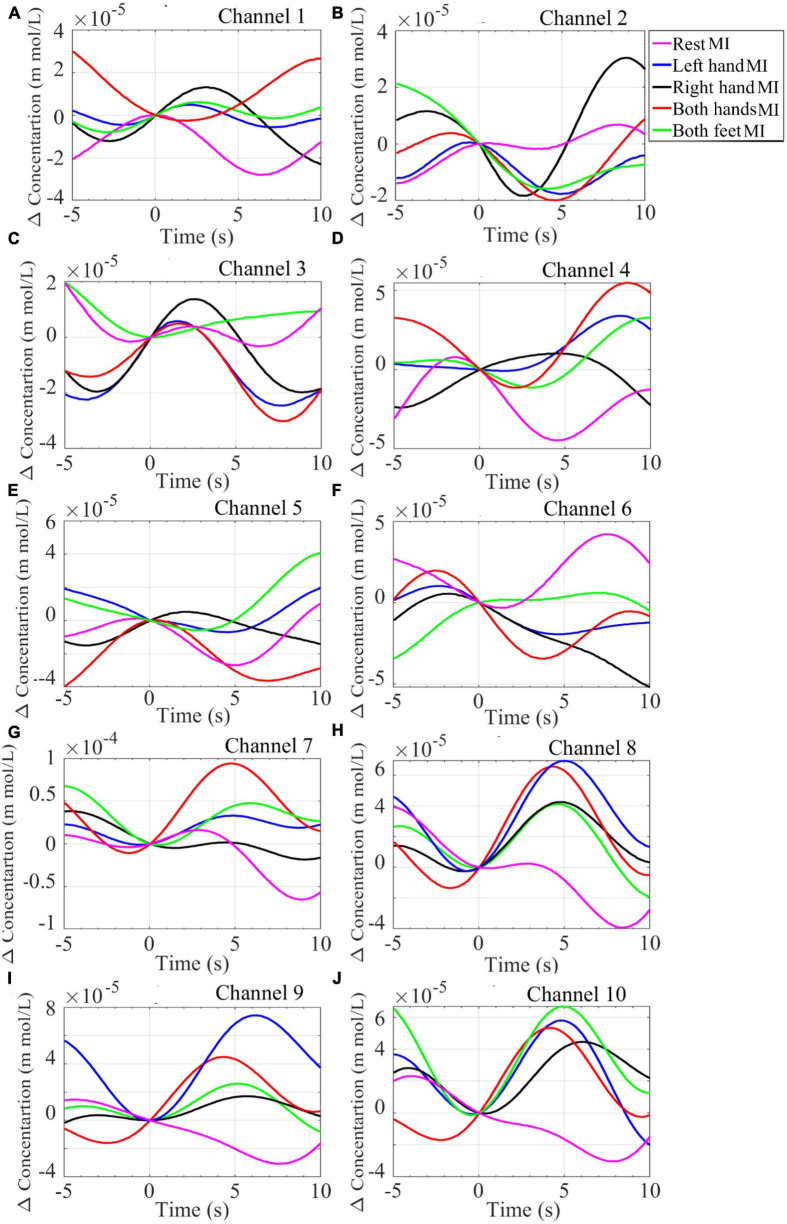
Plot of the grand average of the HbO_2_ concentration from the NIRS signal recorded from 16 participants while performing mentioned MI tasks. Data was collected from channels **(A)** Channel 1, **(B)** Channel 2, **(C)** Channel 3, **(D)** Channel 4, **(E)** Channel 5, **(F)** Channel 6, **(G)** Channel 7, **(H)** Channel 8, **(I)** Channel 9, and **(J)** Channel 10.

Grand averages for all the MI tasks from all the NIRS channels are shown in Figure 8. Channels 1–4 roughly monitor the brain region controlling right-hand movements, channels 5 and 6 feet movements, and channels 7–10 left-hand movements (see Figure 2). Plots of HbO_2_ concentration during the *Right-hand* MI show an increase in value from channels 1–3 while during *Both-hands* MI the values channels 1, 2, 4, 7–10 increase.

HbO_2_ concentration during the *Left-hand* MI increases in channels 7–10 while during the *Both-feet* MI the increase is visible in channel 5. The results from NIRS data as well as the results from the EEG data (ERP and PSD) are consistent with the literature (Shin et al., 2018). It shows that the physiological changes associated with brain activity at specific regions are successfully monitored by our NIRS device and hence it can be combined with tEEG for BCI purposes.

### 3.3. Classification accuracy (offline session)

Features were extracted from the offline session to compare the accuracy across the data-acquiring methods as well as the optimum combination for a BCI application. To extract features, a sliding window of 5 s width and 1-s step size was employed. A matching pursuit (MP) algorithm (Mallat and Zhang, 1993) was directly applied to EEG/tEEG data to extract seven features. To extract five more features fifteen FIR bandpass filters were used to narrow band filter the signals between 5 and 35 Hz (Grosse-Wentrup and Buss, 2008). Each filter has a window of 2 Hz and hence the first filtered from 5 to 7 Hz while the fifteenth filter covered 33–35 Hz. To extract features from these narrow band signals we used signal power, Teager-Kaiser Energy (TKE) operator (Kaiser, 1990), multiclass CSP information theoretic feature extraction (ITFE) (Grosse-Wentrup and Buss, 2008), PSD mean, and amplitude mean.

To extract features by MP from EEG/tEEG data, we prepared a twenty-five Gabor atoms dictionary (see Supplementary Figure 2A). The data within the feature extraction window (5 s window) were compared with a Gabor atoms dictionary. When a match between Gabor atoms and data is found, we calculated the statistical features of matching atoms. Extracted features were mean atom frequency, mean atom amplitude, mean atom phase, mean atom scale, variance of the atom phase, maximum atom amplitude, and the maximum scalar product of each atom with the data within the window.

Seven features were extracted from NIRS measurements: two features by MP, the five other features by ITFE, HbO_2_ amplitude variance, HbO_2_ amplitude mean, HbO_2_ slope mean, HbO_2_ slope mean second derivative. To extract MP features, we created thirty atoms Gabor dictionary (see Supplementary Figure 2B). The extracted MP features are the mean atom amplitude and the maximum scalar product of each atom with the data within the window.

Extracted features were either fed directly to an ensemble classifier (Dietterich, 2000; Maclin and Opitz, 2011) or the feature size was reduced by applying the feature selection method Least Absolute Shrinkage and Selection Operator (LASSO) (Tibshirani, 1996; Tay et al., 2021).

**FIGURE 9:**
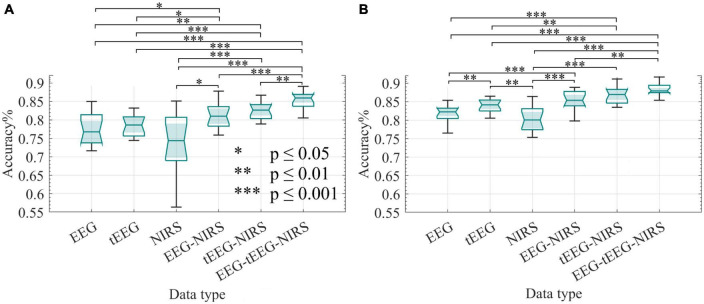
Classification accuracy of BCI measurements collected with NIRS and TCREs during the offline sessions (*n* = 16). Wilcoxon rank-sum test was employed to assess the statistical difference in accuracy between data types. The classifiers were 10-fold cross-validated. **(A)** LASSO feature selection was applied before feeding the selected features to an ensemble classifier. **(B)** All features were fed to an ensemble classifier only. *p ≤ 0.05, ^**^p ≤ 0.01, and ^***^p ≤ 0.001.

Figure 9A displays accuracy calculations resulting from the initial use of LASSO, while Figure 9B shows the accuracy calculated employing an ensemble classifier only. Accuracies above 85% (median around 88%) were achieved when combining all three data sets (EEG + tEEG + NIRS), while accuracies above 83% (median around 87%) were achieved when combining tEEG + NIRS. We used the Wilcoxon rank-sum test to compare the accuracy obtained by different data collection methods namely EEG, tEEG, and NIRS as well as their combinations. Combining NIRS with tEEG or EEG (dual modality) performed significantly better than any single modality as shown in Figures 9A, B. No significant difference was found between NIRS-EEG and NIRS-tEEG. In Figure 9A *p*-values of 8 × 10^–4^ were achieved when EEG was compared with NIRS and 5.6 × 10^–4^ when compared with tEEG. Feature size reduction did not improve the accuracy of comparisons in Figures 9A, B.

### 3.4. Online session

Before commencing the online session for any subject, tEEG data was analyzed with the offline analysis package of the BCI2000 software (Schalk et al., 2004). The analysis showed the active channels during each task. We used the analysis results to determine classifiers for the subsequent online session. During the online sessions, signals were collected with the 10 NIRS channels and eight tEEG channels. Only twelve of the original sixteen subjects participated in the online sessions. Feature extraction of online signals was limited to the identified active channels specified during the offline analysis. Two classifiers were employed, right vs. left and up vs. down.

Twelve features were extracted from tEEG data and seven from the NIRS measurements (with the same methods used for the offline data, see above). Deviating from offline sessions, we used the common spatial pattern (CSP) (Sannelli et al., 2010) instead of ITFE which may be interpreted as a multi-class CSP (Grosse-Wentrup and Buss, 2008; Lotte and Guan, 2011). As mentioned, we only required a two-class classification for the online sessions. A further technical difference between off- and online sessions was the lack of 8 EEG channels for the online montage while the offline one featured 8 EEG and 8 tEEG channels.

**FIGURE 10:**
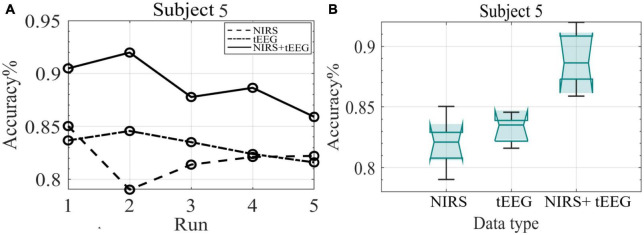
**(A)** Mean classification accuracy of subject 5 data collected during the five online runs. **(B)** Boxplots of the classification accuracy of subject 5 data collected during the online session.

Figure 10A depicts the mean classification accuracies per run for NIRS, tEEG, and NIRS + tEEG data collected from subject 5 during the online session. Figure 10B illustrates boxplots for all five online runs of subject 5. In both graphs, we can see that the combined NIRS + tEEG gave better accuracy compared to a single modality. We can also see that the accuracy of the results with the novel NIRS system is within an acceptable range and their addition to tEEG data gave a significant improvement to the overall accuracy.

**FIGURE 11:**
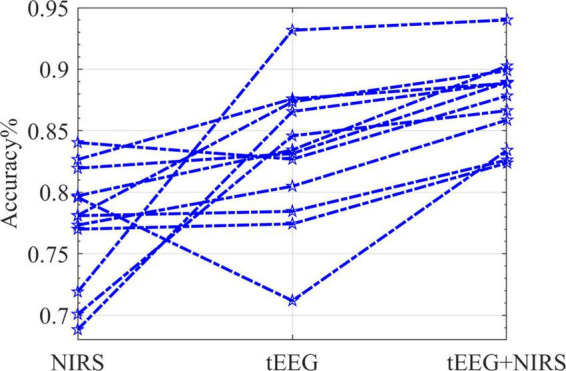
The classification accuracy (five runs per subject) for the online data session (*n* = 12). Each star marks the accuracy 5-fold cross-validated; lines connect one subject’s detailed performance.

Figure 11 illustrates the mean accuracies of the 5 online runs during the online session. For all subjects (*n* = 12), the combined NIRS + tEEG gave overall higher accuracy compared to a single modality. The performances of subjects displayed were quite variable. tEEG data accuracies were higher for some subjects while NIRS measurements showed higher accuracies for others. This could be explained by the different characteristics of each measurement. NIRS is an optical measurement; it is more susceptible to ambient light, skin, and hair color. tEEG is more susceptible to motion artifacts, skin thickness and perspiration.

## 4. Conclusion

Near-infrared spectroscopy application in BCI is relatively new compared to the well-known EEG use. We developed a dual-modal BCI system with two non-invasive modalities at a moderate cost compared to other systems (Scholkmann et al., 2014).

Results from an offline BCI session with 16 subjects showed that their performance fell within a comparable range. It corroborated the novel NIRS system’s capability to efficiently monitor subjects’ brain activity and to assess movement intents. It also revealed differences in accuracy between measurement modalities and their combinations. NIRS performed significantly better when combined with tEEG (vs. EEG). Our decision to utilize the TCRE electrode tEEG to enhance BCI performance thus proved to be correct. Lastly, the offline session allowed us to adjust and individualize classifiers for each subject according to the subject’s performance for the following online session.

Results from the actual BCI experiments, the online session, revealed strong inter-subject differences in the classification accuracies of tEEG and NIRS data. This may be due to different skin/hair colors, skin thickness, as well as other possible physiological and anatomical factors. The general performance shows strong variances between subjects with some performing better due to an inherent capability or higher ability to focus during the sessions or both. However, our NIRS system compared very well with the usual electrical BCI modalities, and it showed even particularly promising results when combined into a hybrid NIRS + tEEG BCI system for all the subjects.

Although the NIRS system was used to monitor the motor areas of the brain, there is no reason why it can’t be efficiently employed to monitor other brain regions of interest as well. The flexibility of montages with optodes arbitrarily fixed on any EEG cap supports further studies. The number of channels may be increased by changing the source-detector alignment map and even add more inexpensive sources or detectors to yield higher channel counts. Our modular system is affordable, portable, lightweight, and can be employed efficiently with other modalities to monitor brain activity at different regions of interest.

## Data availability statement

The raw data supporting the conclusions of this article will be made available by the authors, without undue reservation.

## Ethics statement

The studies involving human participants were reviewed and approved by the Rhode Island IDeA Network for Excellence in Biomedical Research (RI-INBRE). The patients/participants provided their written informed consent to participate in this study.

## Author contributions

RA: conceptualization, visualization, electronics design and assembly, methodology, software, formal analysis, investigation, writing – original draft, and editing. SM: electronics design, review, and editing. AA: review and editing. YB: investigation, review, and editing. KM: funding acquisition, project administration, review, and editing. WB: resources, review, and editing. UH: conceptualization, supervision, project administration, funding acquisition, review, and editing. All authors contributed to the article and approved the submitted version.
