# Supplementary material for: A case for hybrid BCIs: combining optical and electrical modalities improves accuracy
Source: Front Hum Neurosci. 2023 Jun 7;17:1162712. doi: 10.3389/fnhum.2023.1162712 (PMC10282188; doi:10.3389/fnhum.2023.1162712)
Supplement: Supplementary file 1 [file Data_Sheet_1.PDF]

Supplementary Material

1 Supplementary Figures

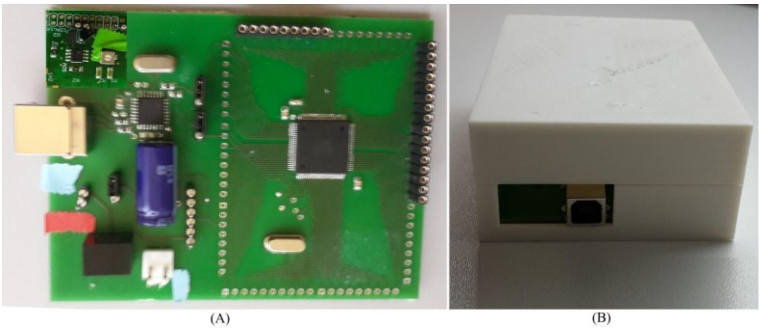

Figure 1: A) NIRS system control circuit board and, B) its 3D printed case.

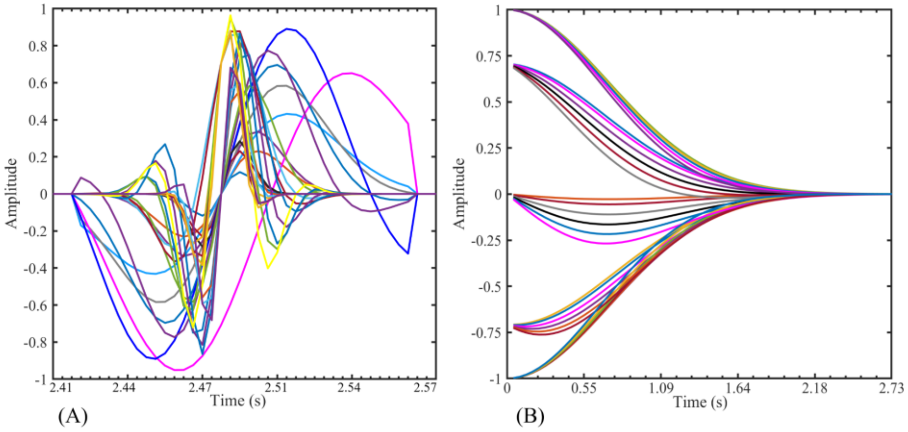

Figure 2: A) The 25 Gabor atoms used for MP feature extraction from EEG/tEEG, B) The 30 Gabor atoms used for MP feature extraction from NIRS measurements.
